# Improving salt tolerance in potato through overexpression of *AtHKT1* gene

**DOI:** 10.1186/s12870-019-1963-z

**Published:** 2019-08-16

**Authors:** Li Wang, Yuhui Liu, Dan Li, Shoujiang Feng, Jiangwei Yang, Jingjing Zhang, Junlian Zhang, Di Wang, Yantai Gan

**Affiliations:** 1 Gansu Provincial Key Laboratory of Aridland Crop Science, Gansu Key Laboratory of Crop Genetic and Germplasm Enhancement, Gansu Agricultural University, Lanzhou, 730070 China; 2 College of Life Science and Technology, Gansu Agricultural University, Lanzhou, China; 3 College of Horticulture, Gansu Agricultural University, Lanzhou, 730070 China; 4 Institute of Soil, Fertilizer and Water-saving Agriculture, Gansu Academy of Agricultural Sciences, Lanzhou, 730070 China; 5 College of Agronomy, Gansu Agricultural University, Lanzhou, 730070 China; 6 College of Resources and Environmental Sciences, Gansu Agricultural University, Lanzhou, 730070 China; 7 Longdong University, Qingyang, 745000 Gansu China; 8 Swift Current Research and Development Centre, Agriculture and Agri-Food Canada, Swift Current, SK S9H 3X2 Canada

**Keywords:** *AtHKT1* gene, *Solanum tuberosum*, K^+^/Na^+^ ratio, Photosynthetic rate, Stomatal conductance, Transpiration rate

## Abstract

**Background:**

Survival of plants in response to salinity stress is typically related to Na^+^ toxicity, but little is known about how heterologous high-affinity potassium transporter (HKT) may help alleviate salt-induced damages in potato (*Solanum tuberosum* L.).

**Results:**

In this study, we used the *Arabidopsis thaliana* high-affinity potassium transporter gene (*AtHKT1*) to enhance the capacity of potato plants to tolerate salinity stress by decreasing Na^+^ content and improving K^+^/Na^+^ ratio in plant leaves, while maintaining osmotic balance. Seven *AtHKT1* transformed potato lines (namely T1, T2, T3, T5, T11, T13 and T15) were compared with non-transgenic control plant at molecule and whole-plant levels. The lines T3 and T13 had the highest *AtHKT1* expression with the tolerance index (an quantitative assessment) being 6.8 times that of the control. At 30 days under 100 and 150 mmol L^− 1^ NaCl stress treatments, the T3 and T13 lines had least reductions in net photosynthetic rate, stomatal conductance and transpiration rate among the seven lines, leading to the increased water use efficiency and decreased yield loss.

**Conclusions:**

We conclude that the constitutive overexpression of *AtHKT1* reduces Na^+^ accumulation in potato leaves and promotes the K^+^/Na^+^ homeostasis that minimizes osmotic imbalance, maintains photosynthesis and stomatal conductance, and increases plant productivity.

**Electronic supplementary material:**

The online version of this article (10.1186/s12870-019-1963-z) contains supplementary material, which is available to authorized users.

## Background

Approximately 30% of irrigated agricultural land is affected by salinity, accounting for 7% of the total arable land on the planet [1, 2]. Salinity affects plant growth through a two-phase physiological challenge, one is osmotic stress and the other is ion toxicity [3]. Osmotic stress reduces plant growth due to decreased water potential [3], whereas sodium ions (Na^+^) congest to a toxic concentration that reduces potassium ions (K^+^) absorption. These challenges cause the disorder of a variety of biological processes in the plant, including physiological characteristics and enzyme activity [4]. In higher plants, remaining a low Na^+^ level and a normal range of K^+^/Na^+^ ratio in cell cytoplasm is necessary to enhance plant salt tolerance [3, 5, 6].

*HKTs* genes, members of the Trk/Ktr/HKT transporter superfamily, are found in plants and microorganisms (Yamaguchi et al., 2013). HKTs have been reported to be active at the plasma membrane level [7], as the HKT transporters exclude Na^+^ from the leaves while increasing K^+^ transportation to resist salt stress [8–11]. *HKTs* genes family splits into subfamilies for different functions. Subfamily I plays a central role in determining the Na^+^ selectivity and K^+^ transportation in higher plants [7, 12–14]. In wheat (*Triticum aestivum* L.), *TmHKT1;5-A* helps retrieve Na^+^ from the xylem and reduces Na^+^ transport to the leaves, and thus enhancing plant tolerance to salt [15]. The overexpression of the *Glycine max GmHKT1* and *GmHKT1;4* genes using the CaMV 35S promoter has been found to affect K^+^ and Na^+^ transport in the roots and shoots, regulate K^+^/Na^+^ homeostasis, and thus improve the tolerance of transgenic tobacco (*Nicotiana tabacum* L.) plants to Na^+^ stress [16, 17]. Similarly, the overexpression of the *Zea mays ZmHKT1;1* gene increases the salt tolerance of the transgenic tobacco plants by increasing the fresh weight and root lengths, raising the K^+^ content and decreasing the Na^+^ content in the roots and shoots [18].

A number of studies have shown that HKT proteins in subfamily II are highly selective and with a tendency of transporting K^+^ in preference to Na^+^ under salt stress. *Puccinellia tenuiflora* PutHKT2;1 is able to take up K^+^ in high NaCl or low K^+^ concentration media [19]. The increased K^+^ homeostasis helps to maintain cytoplasmic properties and thus enhances salinity tolerance in halophytes [20]. In contrast, in *Arabidopsis thaliana*, the HKT homologous protein *AtHKT1*, the member of subfamily I of *HKTs* genes, is found to be a selective Na^+^ transporter in the *Xenopus laevis* oocyte expression system [21]. Localized in the plasma membrane of the xylem parenchyma cells, *AtHKT1* unloads Na^+^ to the xylem parenchyma cells in roots and stems, decreases the Na^+^ transport from the root to stem, and reduces the movement of Na^+^ into leaf cells [22–27]. The strong overexpression of *AtHKT1;1* specifically in the mature root stele may lead to a reduction of Na^+^ transfer from root to shoot, but transgenic *Arabidopsis thaliana* plants with reduced shoot Na^+^ exhibit an increase of salinity tolerance [11]. These results show that the cell-type-specific *AtHKT1*gene expression may serve as an important strategy to resist excessive Na^+^ accumulation in *Arabidopsis* [11]. However, most of the published studies focus on the native *AtHKT1* gene expression and Na^+^ accumulation in plants. Little has been reported on the manner in which the *AtHKT1* gene may influence the physiological, biochemical characterization and K^+^/Na^+^ homeostasis; these possible mechanisms may be involved in the survival of heterologous plants to salt stress [28]. The question remains unanswered whether the *AtHKT1* gene can reduce Na^+^ accumulation, improve K^+^/Na^+^ ratio in heterologous plants and thus enhance plant tolerance to salt stress.

Potato (*Solanum tuberosum* L.) plays an important role in securing global food supply [29] and the crop yield is highly related to the host genetics and crop management practices [30]. Potato plants are moderately sensitive to salinity [31]. Salinity stress during the reproductive period can disturb the normal K^+^/Na^+^ ratio in the leaves [6], negatively affect leaf ultrastructure [32], and decrease leaf photosynthesis [6]. An abnormal K^+^/Na^+^ ratio in potato leaves lowers the movement of carbon from the vegetative tissues to the tubers and reduces the number of tubers and tuber size [33, 34]. However, little has been reported on the manner by which HKTs proteins are involved in the Na^+^ accumulation and maintenance of K^+^/Na^+^ homeostasis in potato.

Although molecular mechanisms underlying the osmotic aspects of salt tolerance remain largely unknown [35], we suggest that a high-affinity potassium transporter (HKT) that regulates plant Na^+^ homeostasis plays a key role in improving salt tolerance in plants through genetic enhancement. In the present study, we transferred the *Arabidopsis thaliana* high-affinity potassium transporter *AtHKT1* gene to the abundantly-grown potato cultivar “Shepody” using the constitutive promoter CaMV 35S. We hypothesize that the overexpression of the *AtHKT1* gene in potato helps to reduce Na^+^ accumulation and balance the K^+^/Na^+^ homeostasis in the leaves, thereby maintains plant osmotic balance under salt stress and advancing plant productivity. To test the hypothesis, we performed a series of measurements, including K^+^ and Na^+^ contents, K^+^/Na^+^ ratio in leaves, key photosynthetic and biophysicochemical traits, antioxidant enzyme activities, plant biomass and tuber yield, and quantified the degree of salt tolerance by developing assessment indices.

## Results

### Plasmid construction and potato transformation

The plasmid pAtHKT1 containing the *AtHKT1* and *NptII* genes was constructed, and both genes were verified using PCR amplification (Additional file 3: Figure S1). After 4 to 5 weeks of culture in a selective shoot medium (MS medium containing 5.71 μmol L^− 1^ IAA and 9.12 μmol L^− 1^ ZT), no shoot was produced in the control line (Additional file 4: Figure S2a). In contrast, green shoots were generated from the transformed microtuber slices (Additional file 4: Figure S2b). Approximately 5 days after the green shoots were transferred to the selective rooting medium (MS medium containing 102.20 μmol L^− 1^ kanamycin and 591.91 μmol L^− 1^ carbenicillin), roots were formed in the transgenic lines (Additional file 4: Figure S2c-1, − 3, and − 4) and no root was formed on the control (Additional file 4: Figure S2c-2). Potato plantlets with well-developed roots were reproduced for further molecular analyses.

### Molecular detection and gene expression analysis

**Fig. 1:**
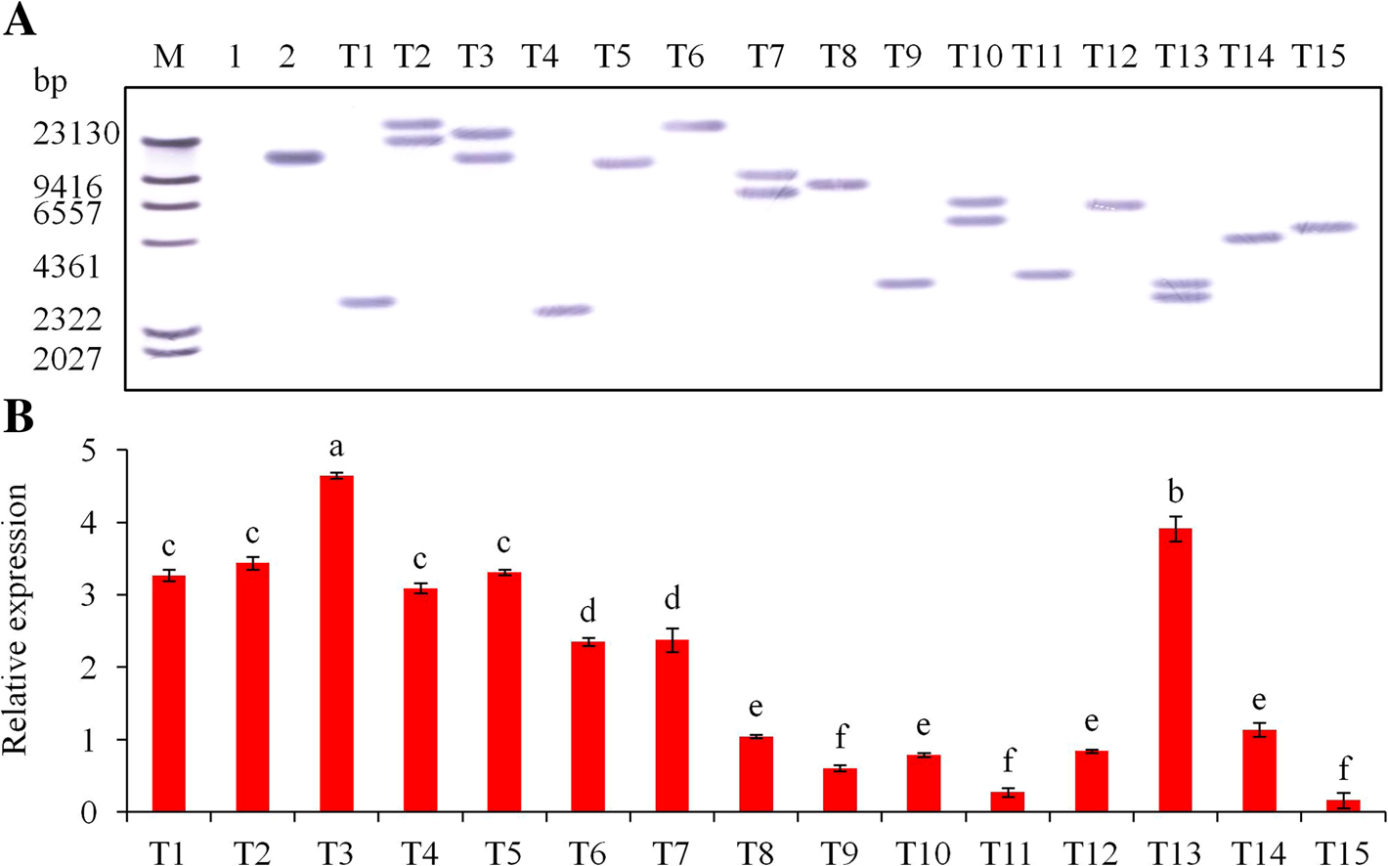
Molecular detection of transgenic potato lines. **a** Southern blot analysis of genomic DNA from PCR-positive transformed potato lines. M - DIG DNA MarkerII; 1 - genomic DNA from the untransformed potato plant as a negative control; 2 - genomic DNA from plasmid AtHKT1 as a positive control; and T1 to T15 are genomic DNA from transgenic potato lines. The genomic DNA was digested with *Hin*d III and hybridized with an *AtHKT1* probe labeled with digoxigenin. And (**b**) expression of *AtHKT1* gene with quantitative real-time PCR in transgenic (T1 to T15) potato plants. Significant differences among means of different transgenic potato plants were determined according to Tukey’s HSD test at *P* < 0.05. The line bars are standard errors (n = 9, i.e., 3 replicates × 3 runs of the experiment)

Using *AtHKT1* gene-specific primers, PCR analysis revealed that the putatively transformed plants had a 1521 bp amplification product and the control plant lacked this product (Additional file 5: Figure S3a). PCR - Southern blot analysis provided additional confirmation of the PCR amplification results, which showed that 20 transformed plants had the 1521 bp *AtHKT1* gene amplification product, while the control plants did not (Additional file 5: Figure S3b). In addition, the Southern blot analysis revealed that the *AtHKT1* gene was integrated into the genomes of the 15 transgenic plant lines (T1, T2, ….T15) with hybridization signals. Five transgenic plant lines without hybridization signals were false positive plant lines. Five of the 15 transgenic lines (T2, T3, T7, T10, and T13) with positive hybridization signals had two copies of the *AtHKT1* gene (Fig. 1a). The expression profiles of the RT-PCR in the leaves also showed that the *AtHKT1* gene was expressed in the 15 transgenic plants, and no transcript was found in the control plants (Additional file 6: Figure S4). These results were confirmed in further detail using qRT-PCR analysis in which *AtHKT1* was expressed in all the transgenic lines (Fig. 1b). There were significant (*P* < 0.05) differences in gene expression among the transgenic lines. The T3 and T13 lines had the greatest *AtHKT1* expression, which were 1.4- to 28.6-fold greater than those of the other transgenic line averages. Based on the quantity of *AtHKT1* gene expression, we selected seven transgenic lines, along with the non-transgenic control, to produce mini-tubers for further studies of the genetic stability and salt tolerance in greenhouse. Two of the lines (T3 and T13) had the highest expression of the *AtHKT1* gene, three had a moderate expression (T1, T2 and T5), and the remaining two had a low expression (T11 and T15).

### Genetic stability and assessment of salt tolerance for the overexpression of the *AtHKT1* gene

**Fig. 2:**
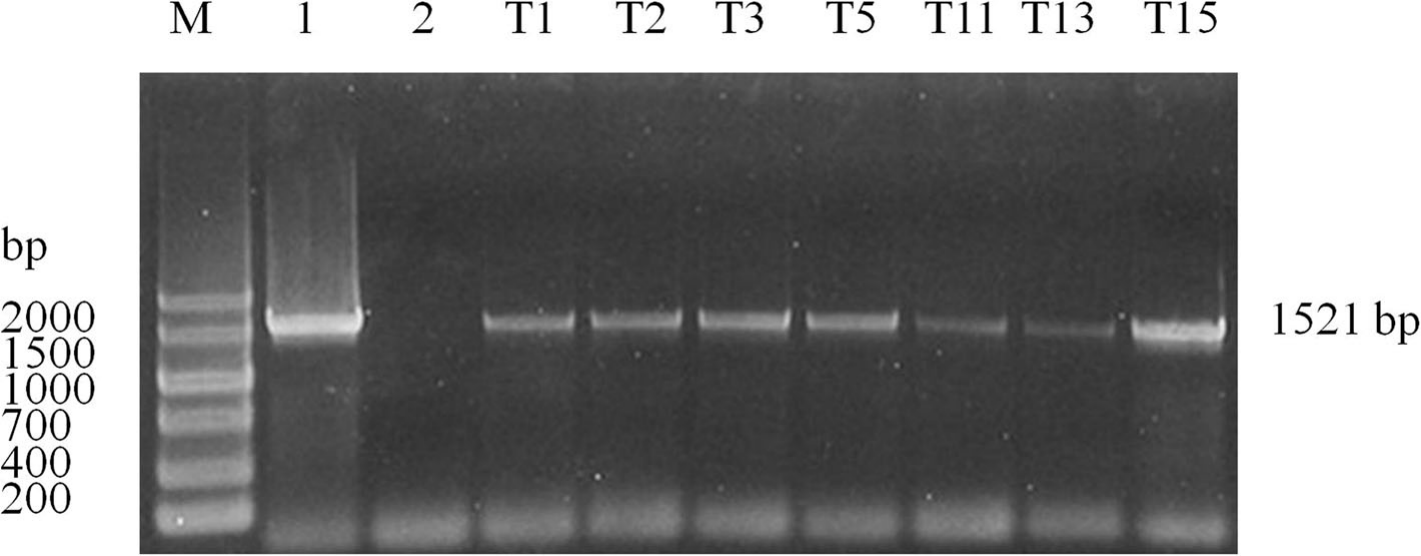
Polymerase chain reaction analysis of the first generation of transgenic potato lines. M - DNA markerV; 1 - plasmid AtHKT1 as a positive control; 2 - untransformed potato plant as a negative control; and T1, T2, T3, T5, T11, T13 and T15 are transgenic potato plants

The PCR amplifications proved that seven (T1, T2, T3, T5, T11, T13, and T15) transgenic lines carried the complete coding sequence of *AtHKT1* gene in the 1521 bp with no specific fragment found in the control plants (Fig. 2). These results showed that the *AtHKT1* gene has stabilized the inheritance in the transgenic potato lines. Thus, the assessment of NaCl tolerance was performed in further detail with the seven of the 15 transgenic lines that overexpressed the *AtHKT1*gene.

Assessment using the six individual growth traits indicated that all the transgenic lines had a significantly (*P* < 0.05) higher salt tolerance than the control plants, as reflected by higher coefficients (Table 1). However, the use of the individual growth traits to test the relative salt tolerance among plants was complicated because each growth trait had a different degree of influence on the magnitude of salt tolerance. For example, there were significant differences in the degree of salt tolerance among the transgenic lines, but the T15 line had a lower coefficient in the tuber yield, a higher coefficient of salt tolerance, and a higher water content (84%) and daily gain in plant height (62%) than the control. T15 and the control plants had an equivalent value for their stem thickness expansion rate, survival rate and leaf area expansion.

**Table 1:** Coefficients of salt tolerance evaluated for each of the six main plant growth variables for the transgenic potato lines over-expression of *AtHKT1* gene and the check cultivar

Genotype^a^	Daily gain in height (%)	Leaf area expansion rate (%)	Stem thickness expansion rate (%)	Relative water content (%)	Yield per plant (%)	Survival rate (%)
CK	49.7 ± 0.7 c	22.0 ± 4.9 bc	42.8 ± 0.3 d	80.0 ± 0.4 e	44.3 ± 1.6 c	52.8 ± 0.7 c
T3	82.2 ± 0.5 a	58.9 ± 1.8 a	73.3 ± 1.5 ab	95.8 ± 0.8 a	89.0 ± 1.1 a	82.6 ± 1.8 a
T13	84.4 ± 1.6 a	50.6 ± 6.6 ab	78.0 ± 0.4 a	95.7 ± 1.2 a	88.4 ± 1.1 a	85.4 ± 1.2a
T1	77.8 ± 1.5 a	37.5 ± 0.0 b	69.0 ± 2.1b	91.6 ± 0.5 b	67.9 ± 1.1 b	77.1 ± 2.1 ab
T2	67.0 ± 3.0 b	25.3 ± 1.8 bc	54.7 ± 1.1 c	87.7 ± 0.3 c	66.6 ± 0.3 b	70.8 ± 2.1 b
T5	64.2 ± 1.4 b	19.8 ± 4.4 bc	45.2 ± 2.0 d	86.6 ± 1.1 cd	62.4 ± 1.0 b	70.1 ± 2.8 b
T11	63.4 ± 3.5 b	22.6 ± 4.3 bc	41.5 ± 0.5 d	82.0 ± 0.9 d	44.3 ± 0.3 c	60.4 ± 1.2 c
T15	61.6 ± 0.9 b	16.2 ± 4.4 c	41.4 ± 1.3 d	83.8 ± 0.2 d	37.3 ± 2.1 d	59.7 ± 0.7 c

^a^CK is the “Shepody” non-transgenic potato cultivar; the rest are transgenic potato lines. Each value ± standard error was obtained from three runs of four treatments with three replicates in each run. Within a column, a higher coefficient indicates higher salt tolerance and the different letters behind the numbers denote significant differences according to Tukey’s HSD test at *P* < 0.05

Rather than using the individual growth traits, we used Eq. (5) in which the six key plant-growth traits were compounded to establish an evaluation index for an quantitative assessment of the salt tolerance. In the compounded evaluation indices (Table 2), the relative weights of each growth trait were calculated. A higher coefficient represents a greater contribution of the growth trait to the salt tolerance index, with coefficients ranging from 0.02 to 1.04. All seven transgenic lines had significantly higher index values than the control (Table 2). The T3 and T13 lines had index values 6.6- to 6.9-fold higher than that of the five other transgenic lines and 1.5- to 6.9-fold higher than control. These quantitative evaluations suggest that T3 and T13 had the greatest salt tolerance, followed by T1, T2, T5 and T11, T15 with moderate tolerance, and the control had the lowest salt tolerance.

**Table 2:** Subordinator functional components and the integrated evaluation index for the transgenic potato lines over-expression of *AtHKT1* and the check cultivar

Genotype^a^	Subordinator functional components^b^						Index^c^	Ranking
	μ(1)	μ(2)	μ(3)	μ(4)	μ(5)	μ(6)		
CK	0.04	0.24	0.11	0.04	0.17	0.02	0.14	7
T3	0.89	1.04	0.89	0.85	0.96	0.92	0.96	1
T13	0.95	0.86	1.01	0.85	0.95	1.00	0.93	2
T1	0.78	0.57	0.78	0.62	0.59	0.75	0.66	3
T2	0.49	0.31	0.42	0.41	0.57	0.56	0.44	4
T5	0.42	0.19	0.17	0.35	0.49	0.54	0.32	5
T11	0.40	0.25	0.08	0.10	0.17	0.25	0.21	6
T15	0.35	0.11	0.07	0.20	0.05	0.23	0.14	7
Weighted value	0.11	0.33	0.19	0.05	0.21	0.11		

^a^CK is the “Shepody” non-transgenic potato cultivar; the rest are transgenic potato lines

^b^The six subordinator functional components are (1) daily gain in plant height, (2)leaf area expansion rate, (3) stem thickness expansion rate, (4)relative water content,(5) tuber yield, and (6) survival rate after salt treatment

^c^The integrated evaluation index was generated using the four subordinator functional components

Using a cluster analysis, we categorized the salt tolerance of the genetic materials into three classes: salt tolerance being high (T3 and T13), medium (T1, T2, and T5), and low (T11, T15 and the control) (Additional file 8: Figure S6). The persistence of salt tolerance in the ‘high’ group was verified further in a controlled-environment experiment in which the different levels of salt stresses (0, 50, 100 and 150 mmol L^− 1^ NaCl) were imposed. At day 45 under 100 mmol L^− 1^ NaCl stress treatment, the control plants (CK) started wilting and showed fewer roots, while the transgenic line T3 plants grew vigorously and showed many roots (Additional file 7: Figure S5). Under 150 mmol L^− 1^ NaCl stress, the control plants’ leaves became yellow and had fewer roots and died, while the transgenic T3 line plants had more roots than control (Additional file 7: Figure S5). Tuber yield measured at harvest did not differ between the transgenic lines and the control under the zero-salt conditions (Table 3). With the NaCl level raised to 50 mmol L^− 1^, the transgenic T3 and T13 lines produced the same yield as those in the zero-salt condition, while the control plants reduced their tuber yield by 15.8% (*P* < 0.05). With the NaCl stress raised to 100 mmol L^− 1^, the transgenic T3 and T13 lines reduced their tuber yield by 11.9%, while the control plant was reduced by 56.7% compared with that in the zero-salt treatment. With the NaCl stress further increased to 150 mmol L^− 1^, the transgenic lines T3 and T13 reduced their tuber yield by 19.9%, but the control plant had a yield reduction by 94.8%.

**Table 3:** Tuber yield (g) per plant of the non-transgenic control (CK) and the transgenic potato plants with high expression of *AtHKT1* gene under no-stress and salt stress

Group^a^	NaCl concentration (mmol L^− 1^)			
	0	50	100	150
CK	242.7 ± 4.1 a^b^	204.3 ± 3.5 bc	105.0 ± 2.9 d	12.7 ± 1.5 e
Transgenic potato	247.5 ± 5.2 a	241.8 ± 4.9 a	218.0 ± 1.7 b	198.2 ± 3.2 c

^a^ CK is the “Shepody” non-transgenic potato cultivar, the other is transgenic potato lines with “High” (T3 and T13) degree of tolerance

^b^ The different letters behind the numbers denote significant differences according to Tukey’s HSD test at *P* < 0.05

With the stress of the NaCl level raised from 0 to 50 mmol L^− 1^, the control plants reduced their root fresh weight by 31.0% and their root dry weight by 18.5%; With the NaCl stress raised to the 100 mmol L^− 1^, the control plants reduced their root fresh weight by 52.2% and their root dry weight by 44.4%. With the NaCl stress increased to 150 mmol L^− 1^, the control plants reduced their root fresh weight by 79.3% and root dry weight by 81.5%, and the transgenic plants exhibited weight reductions of 40.7 and 43.2%, respectively. The control plants reduced the weights of their roots more significantly than the transgenic plants. The shoot fresh and dry weights responded similarly to the treatments as the root traits: with salt stress (NaCl level) raised from 0 to 150 mmol L^− 1^, the control plants reduced their shoot fresh weight by 83.3% and shoot dry weight by 82.2%, while the transgenic plants reduced by 36.8 and 34.2%, respectively (Table 4).

**Table 4:** Fresh and dry weight of potato of the non-transgenic control (CK) and the transgenic plants with high expression of *AtHKT1* gene under no-stress and salt stress

Weight (g)	Group^a^	NaCl concentration (mmol L^−1^)			
		0	50	100	150
Root fresh weight	CK	18.4 ± 0.5 b^b^	12.7 ± 0.7 c	8.8 ± 0.6 d	3.8 ± 0.5 e
	Transg.	23.1 ± 1.3 a	19.1 ± 0.4 b	15.9 ± 0.5 bc	13.7 ± 0.4 c
Root dry weight	CK	2.7 ± 0.1 c	2.2 ± 0.1 d	1.5 ± 0.0 e	0.5 ± 0.0 f
	Transg.	3.7 ± 0.1 a	3.1 ± 0.0 b	2.6 ± 0.0 c	2.1 ± 0.0 d
Shoot fresh weight	CK	184.3 ± 3.9 b	157.3 ± 0.8 c	49.7 ± 1.3 e	30.7 ± 1.4 f
	Transg.	208.5 ± 5.0 a	215.6 ± 5.9 a	160.9 ± 2.3 c	128.0 ± 3.5 d
Shoot dry weight	CK	46.0 ± 0.7 b	31.6 ± 0.7 c	15.3 ± 0.7 d	8.2 ± 0.2 e
	Transg.	51.4 ± 0.5 a	52.6 ± 0.4 a	43.6 ± 0.2 b	33.8 ± 1.0 c

^a^CK is the “Shepody” non-transgenic potato cultivar, the other is transgenic potato lines with “High” (T3 and T13) degree of tolerance

^b^The different letters behind the numbers denote significant differences according to Tukey’s HSD test at *P* < 0.05

### Na^+^, K^+^ contents and K^+^/Na^+^ to salt stress

**Fig. 3:**
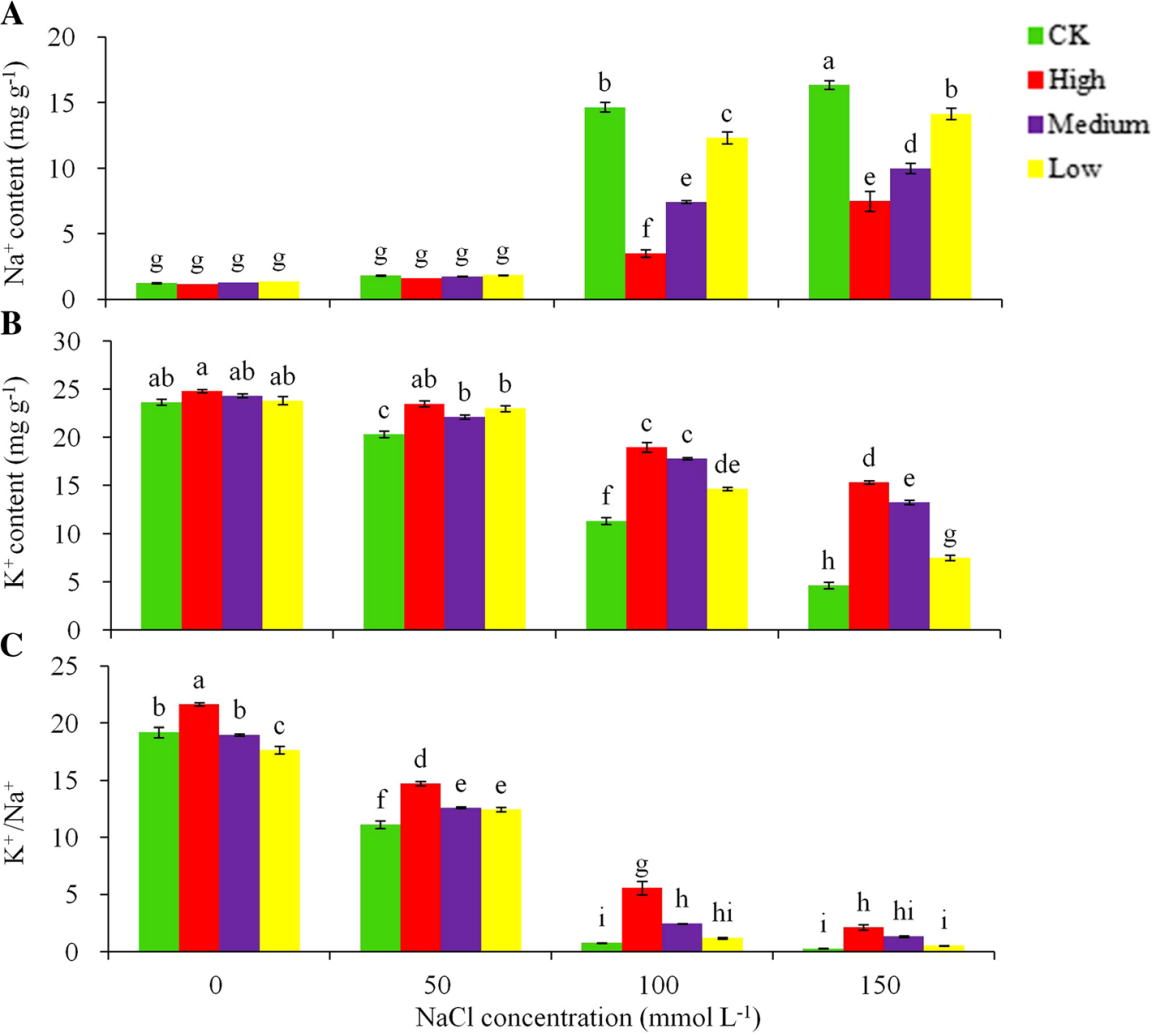
Effects of different levels of NaCl stress on Na^+^ (**a**), K^+^(**b**) and the K^+^ /Na^+^ (**c**) in leaves for the non-transgenic control (CK) and the transgenic lines with with ‘High’ (T3 and T13), ‘Medium’ (T1, T2 and T5), ‘Low’ (T11 and T15) degree tolerance (as ranked in Table 2). Significant differences among means over the NaCl-stress treatments were determined according to Tukey’s HSD test at *P* < 0.05. The line bars are standard errors (*n* = 9, i.e., 3 replicates × 3 runs of the experiment)

To examine the effect of overexpression of the *AtHKT1* gene on the plant tolerance to salt, we measured the K^+^ and Na^+^ contents and K^+^/Na^+^ ratios in the leaves under different levels of NaCl stresses (Fig. 3). K^+^ contents in the leaves increased significantly (*P* < 0.05) with the increase in the NaCl stress for all the genotypes assessed in this study (Fig. 3a). With the increased salt stress, the control plants increased their Na^+^ content remarkably more than the transgenic lines in the leaves (Fig. 3a). By day 30 under the 50 mmol L^− 1^ NaCl stress treatment, the Na^+^ contents were 1.6–1.8 mg g^− 1^ DW in the leaves in the transgenic plants, and 2.1 mg g^− 1^ DW in the control plant. These did not differ significantly. Under 150 mmol L^− 1^ NaCl stress treatment, the control plant Na^+^ contents in the leaf were 2.2- fold greater than those of the transgenic lines. The plant tissues exhibited reverse effects for the K^+^ contents compared to the Na^+^ content (Fig. 3a vs. b). The magnitude of the reduction in K^+^ content varied substantially among the genotypes with the control plants experiencing a reduction significantly greater than the transgenic plants (Fig. 3b). By day 30 of the NaCl treatments, the K^+^ content in the leaves in the transgenic lines plant decreased significantly less than the control plants as the NaCl stress level increased. Significant (*P* < 0.05) differences in the K^+^/Na^+^ ratio were found between the treatments in the leaves (Fig. 3c). The control plants decreased the K^+^/Na^+^ remarkably compared to the transgenic lines under 100 and 150 mmol L^− 1^ NaCl stress treatments.

### Photosynthetic responses to salt stress

**Fig. 4:**
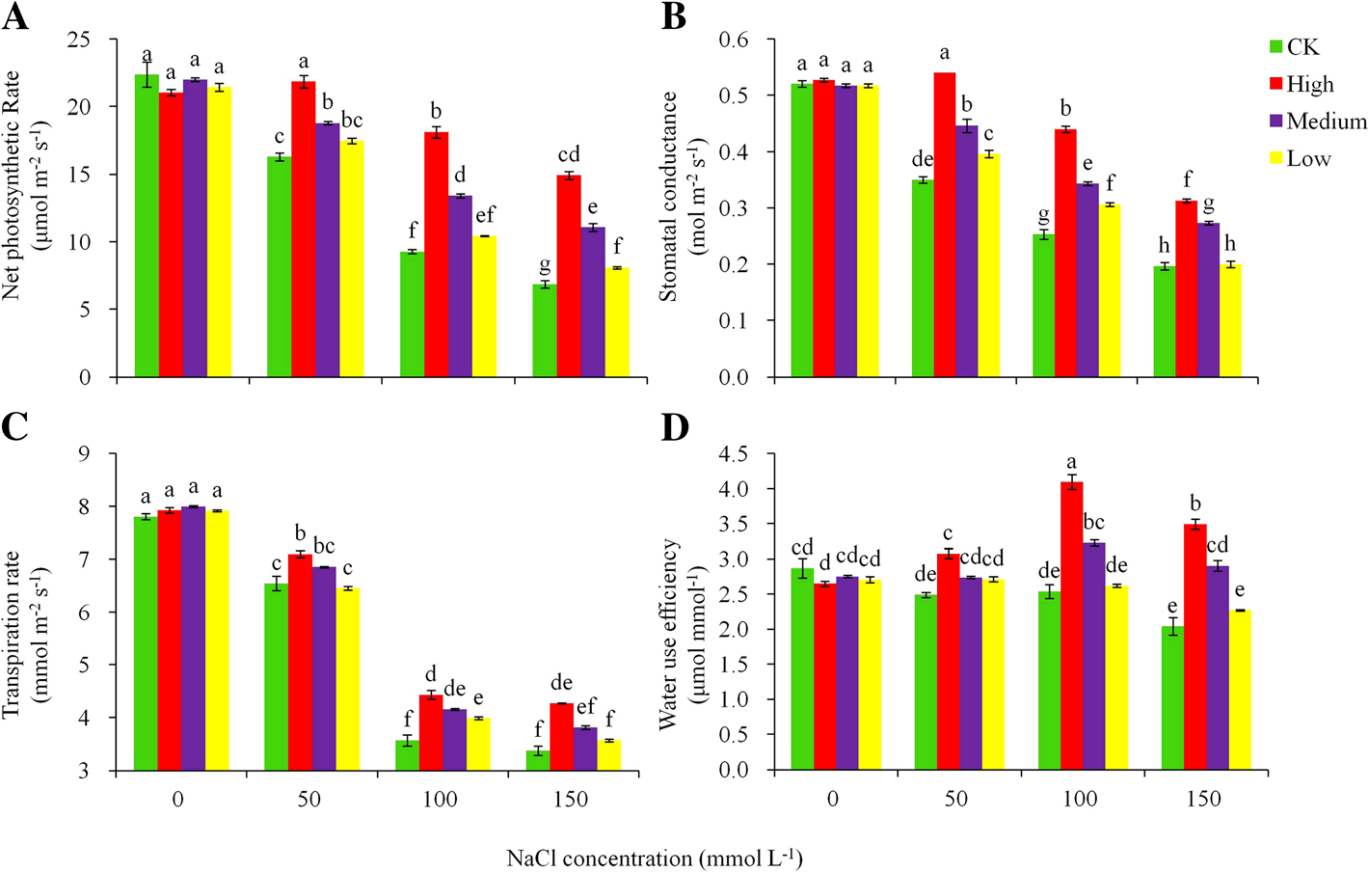
Effects of different levels of NaCl stress on (**a**) net photosynthetic rate, (**b**) stomatal conductance, (**c**) transpiration rate, and (**d**) instantaneous water use efficiency from gas exchange for the non-transgenic control (CK) and the transgenic lines with ‘High’ (T3 and T13) expressions (as ranked in Table 2). Significant differences among means over the NaCl-stress treatments were determined according to Tukey’s HSD test at *P* < 0.05. The line bars are standard errors (n = 9, i.e., 3 replicates × 3 runs of the experiment)

NaCl stress affected the photosynthetic activities in potato. Higher NaCl stress reduced the net photosynthetic rate (Pn, Fig. 4a), stomatal conductance (Gs, Fig. 4b), transpiration rate (Tr, Fig. 4c), and instantaneous water use efficiency (WUE, Fig. 4d) in all the genotypes evaluated. The magnitude of the reduction was significantly higher in the control than in the transgenic lines at the higher salt levels. At day 30 in the NaCl stress treatments, the control plant reduced the Pn by 45.0% under 100 mmol L^− 1^ NaCl stress and 61.6% under 150 mmol L^− 1^ NaCl stress, compared with the zero-salt treatment, while the corresponding decreases in the transgenic lines were 13.7 and 28.9% (Fig. 4a); Gs and Tr displayed a similar trend of response to the NaCl stress in which the control plants reduced the Gs by 32.7% under 50 mmol L^− 1^ NaCl stress, 51.3% under 100 mmol L^− 1^ and 62.2% under 150 mmol L^− 1^ NaCl stress, compared with the zero-salt treatment (Fig. 4b); the decreasing values in the Tr were 16.7, 53.8 and 56.8%, respectively (Fig. 4c). These defense responses led to an increased WUE with salt stress in the transgenic lines in the ‘high’ and ‘medium’ tolerant groups compared to the control (Fig. 4d).

### Physiological and biochemical responses and antioxidant enzyme activities to salt stress

**Fig. 5:**
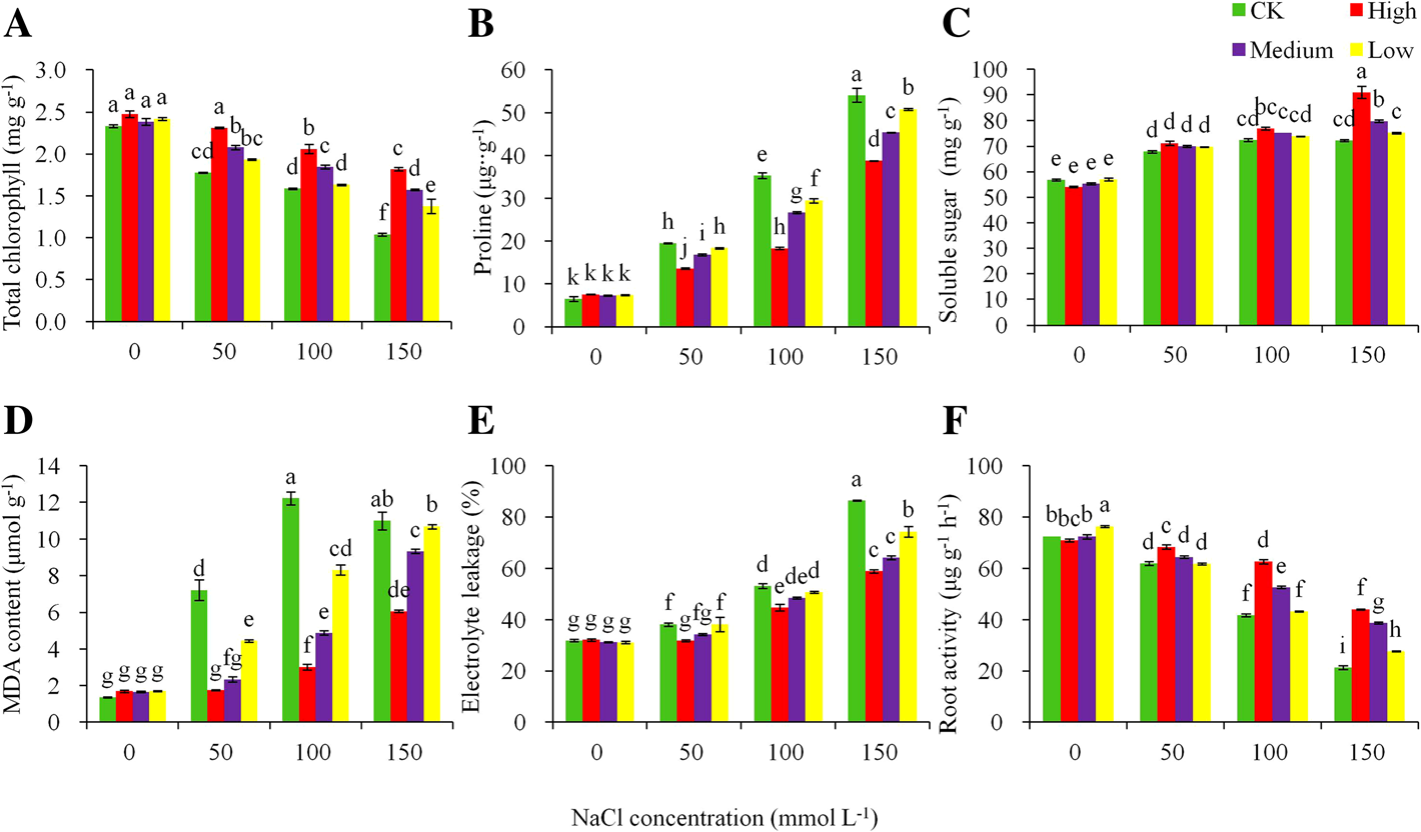
Regressions of various levels of NaCl stress on (**a**) total chlorophyll content, (**b**) proline content, (**c**) soluble sugar, (**d**) malondialdehyde (MDA) content, (**e**) electrolyte leakage, and (**f**) roots activity for the non-transgenic control (CK) and the transgenic lines with ‘High’ (T3 and T13) expressions (as ranked in Table 2). All the regressions were significantly differences at *P* < 0.05

Physiological and biochemical responses and antioxidant enzyme activities in the leaves of potato plants were tested at each of the four NaCl levels, including total chlorophyll content, proline content, soluble sugar content, MDA, electrolyte leakage, root activity, SOD, POD, and CAT. The increased NaCl stress significantly lowered the total chlorophyll content (*P* < 0.05) in both the transgenic and control plants (Fig. 5a). The reduction was more dramatic in the control than in the transgenic lines. For each unit of salt increase (in the range of 0 to 150 mmol L^− 1^ NaCl), the control plants lowered their chlorophyll content by 0.0081 mg g^− 1^ fresh weight, nearly double that in the transgenic lines. The leaf proline content showed a reverse trend to the chlorophyll content: with the increase of NaCl stress, the transgenic lines plants increased their proline content by 0.3175 mg g^− 1^ for each unit of salt increase, with the control plants increasing 60% more than transgentic lines (Fig. 5b). The soluble sugar contents were significantly (*P* < 0.05) different among the treatments with the transgenic lines raising their soluble sugar contents more than the control at the high salt stress level (Fig. 5c).

Significant effects of the salt treatments on the MDA content were found, with the magnitude of the effect varying between the control and the transgenic lines. The MDA in the high salt tolerance transgenic plants was less affected by NaCl compared with the other transgenic lines (Fig. 5d). With each unit of salt increase, the high salt tolerance transgenic plants raised their MDA content by 0.0288 μmol g^− 1^ of fresh weight, while the MDA increase in the control was significantly greater than that in the transgenic plants. At the 100 mmol L^− 1^ NaCl stress, the MAD content in the control plants was 8.7-fold higher than that in the transgenic plants. Electrolyte leakage in the leaves followed a similar trend of salt stress effect as the effect on MDA, and the overall differences were small between the control and the transgenic plants (Fig. 5e).

‘Root activity’ was assessed using a quantitative approach in this study. All the genotypes significantly reduced their root activity (*P* < 0.05) as the NaCl concentration increased (Fig. 5f). For each unit of salt increase, the control plant decreased root activity by 0.3475 μg g^− 1^ h^− 1^, which was more than double the decrease (0.1734) in the transgenic lines. At the highest salt concentration (150 mmol L^− 1^) level, the root activity of the transgenic lines was twice higher that in the control plants.

**Fig. 6:**
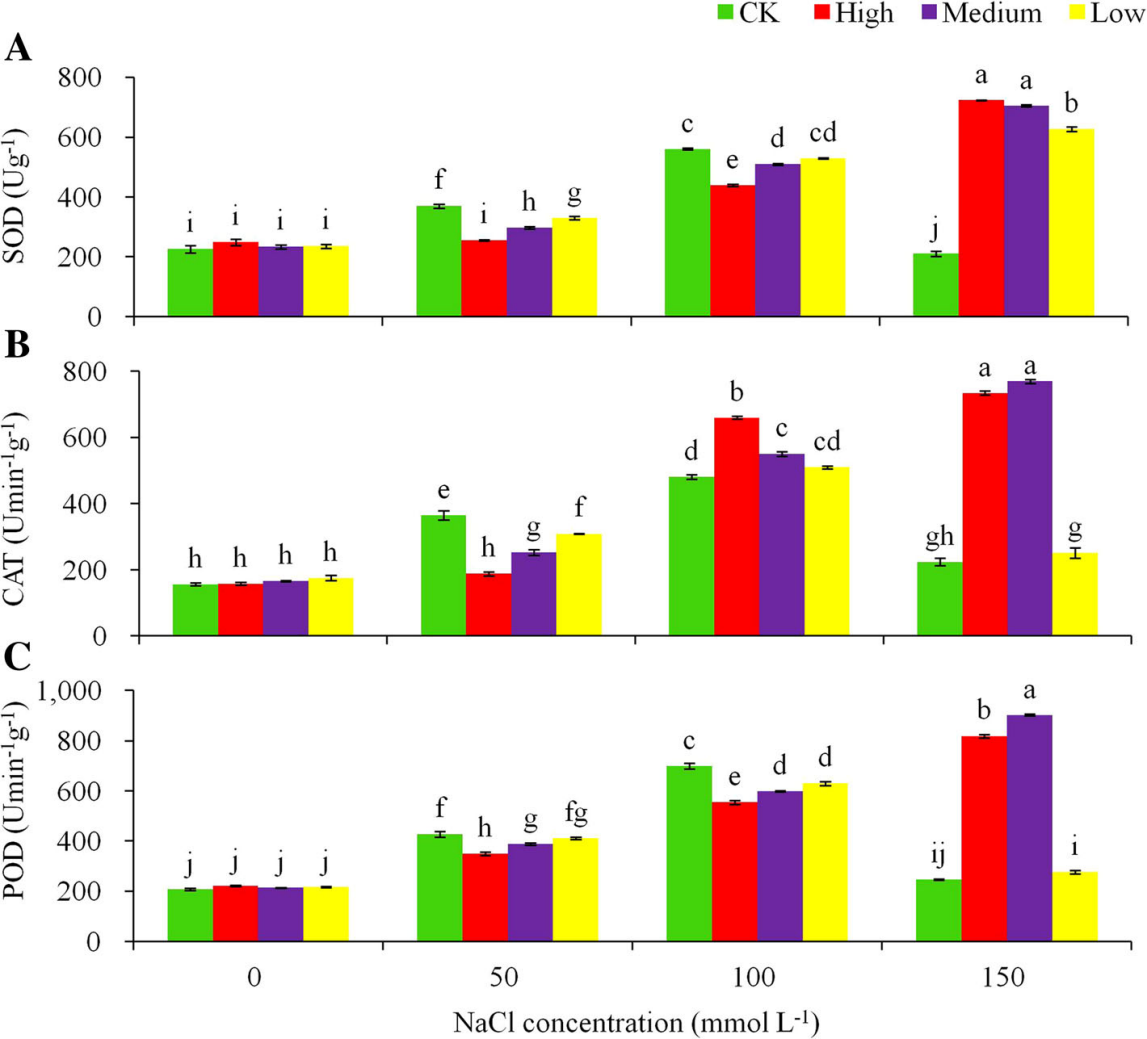
Effects of various levels of NaCl stress on (**a**) superoxide dismutase (SOD), (**b**) catalase (CAT) and (**c**) peroxidase (POD) for the non-transgenic control (CK) and the transgenic lines with ‘High’ (T3 and T13) expressions (as ranked in Table 2). Significant differences among means over the NaCl-stress treatments were determined according to Tukey’s HSD test at *P* < 0.05. The line bars are standard errors (*n* = 9, i.e., 3 replicates × 3 runs of the experiment)

In addition, significant differences in the response to salt stress were found between the control and the transgenic plants in antioxidant enzyme activities, including SOD (Fig. 6a), CAT (Fig. 6b), and POD (Fig. 6c). The NaCl stress promoted enzymatic activities in general, but the trend of the response differed between treatments. The transgenic lines had significantly higher enzymatic activities than the control plants at the 150 mmol L^− 1^ NaCl stress. Inconsistent responses were found at the other salt stress levels between the transgenic lines and the control.

**Fig. 7:**
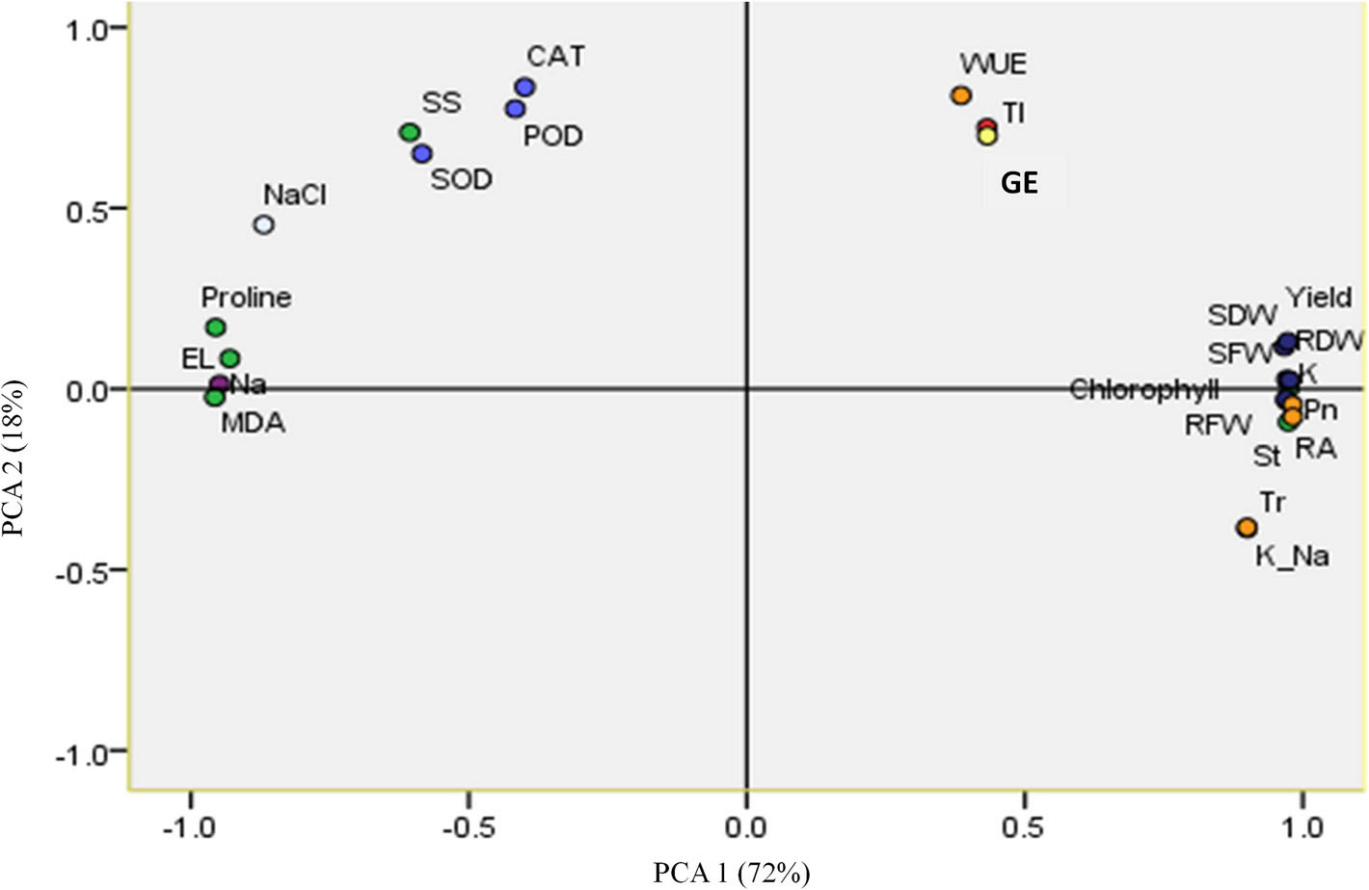
Principle component analysis of transgenic plant physiological and biochemical traits, antioxidant enzyme activities, photosynthetic traits, yield-related variables, gene expression and salt tolerance evaluation index. Physiological and biochemical traits [green symbols, including chlorophyll (total chlorophyll), EL (electrolyte leakage), MDA (malondialdehyde), proline, SS (soluble sugar), RA (roots activity)]; antioxidant enzyme activities [light blue symbols, including SOD (superoxide dismutase), CAT (catalase), POD (peroxidase)]; photosynthetic traits [orange symbols, including Pn (net photosynthetic rate), Tr (transpiration rate), St (stomatal conductance), WUE (water use efficiency)]; Na^+^, K^+^, K/Na (purple symbols); yield-related variables (dark blue symbols, including yield, SFW (shoot fresh weight), SDW (shoot dry weight), RFW (root fresh weight), RDW (root dry weight); NaCl (NaCl concentration, white symbol); TI (tolerance index, red symbol); GE (Gene expression, yellow symbol)

The PCA with all the physiological and biochemical traits, including antioxidant enzyme activities, photosynthetic parameters, yield-related variables, *AtHKT1* gene expression and salt tolerance evaluation index captured the majority of variance in the dataset, with PC axes 1 and 2 explaining 72 and 18% of the total variance, respectively (Fig. 7, Additional file 2: Table S2). Overall, there were positive correlations among *AtHKT1* gene expression (GE), salt tolerance evaluation index (TI), plant photosynthetic traits, yield-related variables, K^+^ content and total chlorophyll content. Especially the GE was highly associated with TI (GE and TI points are almost coincided, Fig. 7), the correlation value was 0.91at *P* < 0.01. Water use efficiency (WUE) was highly positively correlated with GE and TI. Further, we observed that yield-related variables, such as tuber yield, shoot fresh weight (SFW), shoot dry weight (SDW), root fresh weight (RFW) and root dry weight (RDW), were closely clustered with total chlorophyll content, roots activity (RA), K^+^/Na^+^ and net photosynthetic rate (Pn), transpiration rate (Tr), stomatal conductance (St). The *AtHKT1* gene expression was negatively associated with Na^+^ content, electrolyte leakage (EL), malondialdehyde (MDA) and proline. However, no effect among *AtHKT1* gene expression, soluble sugar (SS) and antioxidant enzyme activities (SOD, POD and CAT) was detected.

## Discussion

High-affinity potassium transporters (HKTs) have been known to be critical components of salt stress resistance in plants [36–38]. Many studies have analyzed the gene expression of the native HKTs in species, such as *Pongamia pinnata* [37] and *Triticum aestivum* [5, 38–40]. However, little has been reported on the manner in which the HKTs may be involved in reducing Na^+^ accumulation and adjusting the K^+^/Na^+^ homeostasis in heterologous plants under salt stress. In the present study, we transferred the *AtHKT1* gene into an abundantly-grown potato cultivar and detected that the transgene was integrated into the genome. The quantity of gene expression differed among the transgenic plant lines that were evaluated using molecular tools – PCR, PCR-Southern blot, Southern blot, RT- PCR and qRT-PCR. Our results revealed that the transgenic potato lines with high *AtHKT1* gene expression decreased Na^+^ accumulation, maintained the K^+^/Na^+^ homeostasis and reduced plant cell damage.

We found a remarkable difference in the *AtHKT1* gene expression among the transgenic lines, with two of the lines (T3 and T13) having the highest *AtHKT1* expression. In the scientific literature, the gene expression of the native HKTs in subfamily I is shown to play an important role in enhancing salt tolerance. For instance, the xylem sap Na^+^ concentration and Na^+^ delivery from root to shoot were increased because of the loss of the *ZmHKT1* function, suggesting that *ZmHKT1* improves plant salt tolerance by dropping out Na^+^ from the xylem sap and leaf Na^+^ exclusion [5]. The *OsHKT1;5* transcript was increased under salt stress in the roots and the basal nodes in *Oryza sativa* plants [41]. The silencing of *ScHKT1;2* and *SlHKT1;2* changed the leaf K^+^/Na^+^ ratio and made hypersensitivity to salinity in *Solanum lycopersicum* plants [42]. The increased relative expression of *HKT1* in the roots of *Fragaria ananassa* has been found to be correlated with high tolerance to salinity [43]. In previous studies, *AtHKT1* gene function is found to vary in different *Arabidopsis thaliana* accessions. The gene *AtHKT1* drives natural variation in the adaptation of *Arabidopsis thaliana* to salinity, and the high expression of the shoot *AtHKT1* determines the salt tolerance of the coastal accession [44]. Møller et al. (2009) reported that the specific overexpression of *AtHKT1;1* in the mature root stele increased salinity tolerance, and overexpressing *AtHKT1* in the cortex and epidermis decreases shoot Na^+^ accumulation and improves salt tolerance [11]. Most studies show that cell-type-specific *HKTs* gene expression reduces Na^+^ accumulation in plants, while some other studies reveal that the constitutive overexpression of *HKTs* gene of subfamily I can improve plant salt tolerance [14]. For instance, the overexpression of the *Glycine max GmHKT1* gene significantly affected K^+^ and Na^+^ transport in roots and shoots, regulated Na^+^/K^+^ homeostasis and enhanced the tolerance of transgenic tobacco plants to salt stress [17]. The constitutive overexpression of *GmHKT1;4* significantly enhanced the tolerance of transgenic tobacco plants to NaHCO_3_ and NaCl stresses by accumulating more K^+^ and less Na^+^ under salt stress compared with non-transgenic plants [16]. Our present study add new information to the scientific literature that the heterologous *AtHKT1* overexpression in potato is highly negatively associated with Na^+^ accumulation and positively associated with the K^+^ content and K^+^/Na^+^ homeostasis in leaves; this provides some evidence that the two HKT subfamilies offers an important division of function in salt tolerance [14, 45]. *HKTs* gene of subfamily I particular residue plays a central role in determining the Na^+^ selectivity of the transporter [14, 45], as those proteins are involved in the process of salt tolerance development in potato.

In many studies, researchers attempt to evaluate the degree of salt tolerance using individual growth variables, resulting in a biased ranking of the tolerance [46–48]. In our study, we used an salt tolerance evaluation index, in which a quantitative ranking of the tolerance to salt stress was determined using the six key performance traits. The *AtHKT1* gene expression was highly positively associated with salt tolerance evaluation index, indicating that the *Arabidopsis thaliana* high-affinity potassium transporter-encoding gene *AtHKT1* plays a significant role in improving plant growth under salt stress through promoting a high level of tolerance to salt stress.

Damage to plants due to high salinity results in hydraulic failure, stomatal closure, reduced photosynthesis and enzyme activity, and decreased crop yield mainly due to osmotic imbalance [35]. Reducing Na^+^ accumulation, improving K^+^ accumulation and thus maintaining K^+^/Na^+^ homeostasis in the shoot can maintain the osmotic balance, which is identified as one of the major strategies for enhancing salinity tolerance [39]. These processes may alleviate osmotic imbalance. Additionally, the avoidance of salt-induced hydraulic failure via stomatal closure may alleviate possible structural damage to plant photosynthesis and a cascade of downstream effects. In the present study, our measurements on the Na^+^, K^+^ content and K^+^/Na^+^ ratios in leaves enabled the evaluation of the osmotic differences between transgenic plants with the *AtHKT1* expression and non-transgenic control under salt stress. The significantly lower Na^+^ content and higher K^+^/Na^+^ in the transgenic plants with higher *AtHKT1* expression indicate that the transgenic plants did not suffer Na^+^ poisoning under severe salt stress as much as did the control plants. The *AtHKT1* gene expression in potato had a negative correlation with Na^+^ accumulation and a positive correlation with the K^+^ content and K^+^/Na^+^ ratio in leaves. These observations demonstrate that the constitutive overexpression of *AtHKT1* gene in transgenic potato can help to reduce the Na^+^ content and maintain K^+^/Na^+^ homeostasis in leaves, leading to enhanced salt tolerance.

The *AtHKT1* overexpression appears to confer multiple functions in potato, as the *AtHKT1* expression is positively correlated with plant photosynthetic traits (net photosynthetic rate, transpiration rate, stomatal conductance), yield-related variables (shoot fresh weight, shoot dry weight, root fresh weight and root dry weight), total chlorophyll content and root activity (Fig. 7). The *AtHKT1* gene expression was negatively associated with electrolyte leakage (EL), malondialdehyde (MDA) and proline, with no or little association with soluble sugar and antioxidant enzyme (SOD, POD and CAT) activities. These associations suggest that the expression of *AtHKT1* genes offered the capacity of helping decrease cell damage under salt stress, maintain the photosynthetic activities, and balance soluble sugar content and antioxidant enzyme activities. This phenomenon was supported by the fact that at the high level of salt stress (150 mM NaCl) concomitant protein degradation occurred in non-transgenic control plant that lost antioxidant enzyme activity, while the high salt-tolerant transgenic lines still had strong SOD, CAT and POD enzyme activity. Consequently, the plants with the overexpression of *AtHKT1* gene minimized yield loss and improved water use efficiency under salt stress.

## Conclusions

This research tested the hypothesis that the overexpression of the *AtHKT1* in the potato maintains plant osmotic balance under salt stress, thereby advancing plant productivity. Our results demonstrated that under salinity stress, the transgenic plants with a higher expression of *AtHKT1* accumulated less Na^+^ in plant leaves, decreased the toxicity of Na^+^, maintained a high K^+^/Na^+^ ratio in leaves, and improved key physicochemical activities. The enhanced physiochemical activities ensure the continuous CO_2_ uptake and nutrient supplies, thus reducing the harmful impact of salt stress. The constitution of the over-expression of *AtHKT1* in potato plants advanced their water status under salt stress that improved a conversion from osmotic imbalance to osmotic balance. In addition, the over-expression of the *AtHKT1* in heterologous plants extended the ability to maintain plant growth and yield even when under high salt stress.

## Methods

### Plant materials and vector construction

This study was performed at the Gansu Provincial Key Lab for Aridland Crop Sciences, Gansu Agricultural University in 2012 and 2013. The microtubers of the potato cultivar ‘Shepody’ were used in all the tests, which were from Gansu Key Laboratory of Crop Genetic and Germplasm Enhancement. The culture methods were the same as those described in a previous publication [49].

The 1521 bp cDNA of the *AtHKT1* gene (AY685182) isolated from *Arabidopsis thaliana* was cloned into the *Bam*H I-*Sac* I site of the plasmid pBI121 containing the CaMV 35S promoter, thus replacing the *β-glucuronidase* (*GUS*) gene with the *AtHKT1* gene. The recombinant plasmid pAtHKT1 was introduced into *Agrobacterium tumefaciens* strain *LBA4404* using the freeze-thaw method [50]. The presence of the plasmid was verified using restriction enzyme digestion and PCR amplification.

### Transformation, PCR, PCR-southern, southern blot and reverse transcription RT-PCR analysis

The detailed procedures of the potato transformation, plant genomic DNA extraction, total RNA extraction, reverse transcription, PCR-Southern, Southern Blot and RT-PCR were conducted as described previously [49]. In brief, the slices of the ‘Shepody’ microtubers were used as the receptor for *Agrobacterium*-mediated transformation. The *AtHKT1* gene was amplified using the forward primer 5′- CGGGATCCATGGACAGAGTGGTGGCAAAAATAG -3′(1 - 31 bp) and the reverse primer 5′- CGGAGCTCTTAGGAAGACGAGGGGTAAAGTATCC - 3′(1490 - 1521 bp), which generated an expected PCR product fragment of 1521 bp. The amplification was performed in a thermal cycle (UNO II, Biometra) programmed for one cycle of 1 min at 94 °C, followed by 35 cycles of 50 s at 94 °C, 60 s at 55 °C, and 90 s at 72 °C. A final extension step was performed for 10 min at 72 °C.

### Gene expression assays using quantitative real-time PCR

Total RNA was extracted from the plantlets of fifteen transgenic potato lines and a non-transgenic control. The RNA quality, reverse transcription and qRT-PCR amplification were performed as described by Wang [49]. *efla* was the internal control gene with the forward primers 5′-CAA GGATGA CCC AGC CAA G − 3′ and the reverse primers 5′-TTCCTT ACC TGAACGCCT GT-3′. The forward and reverse primers of the *AtHKT1*gene were 5′ - ATC TGG CTC CTA ATC CCT CAA - 3’and 5′ - CCG TCA CTC CAA GAA GAA CAC - 3′, respectively. Each Quantitative Real-Time PCR was independently repeated three times. A blank control was included in which no cDNA was added to the reaction mixtures. After each reaction, melt curve analysis was used to verify the specificity of amplification, and the relative expression levels were calculated by 2^−ΔΔCt^.

### Physiochemical evaluations, yield and biomass of transgenic potato transformed with the *AtHKT1* gene

In controlled-environment greenhouses, mini-tubers of the *AtHKT1* transgenic potato lines were evaluated for their levels of stress resistance. The mini-tubers were outputted from test-tube seedlings as previously described [51]. In brief, test-tube seedlings were transferred in a plate (30 × 60 cm and 10 cm in height) filled with vermiculite [(NH_4_)_2_HPO_4_ 26.7 g kg^− 1^, KH_2_PO_4_ 13.3 g kg^− 1^] under room temperatures. The vermiculite moisture was maintained at 70–80% field capacity. A nutrient solution at 100 ml MS (pH 6.0) was added to each plate every seven days. Mini-tubers were harvested at maturity and stored at 4 °C for 3 months. After recovery from dormancy, each mini-tuber was planted in a pot (32 cm in diameter and 25 cm in height) filled with 50% loessial soil and 50% vermiculite (v/v) under room temperatures. All pots were watered daily to maintain the vermiculite moisture at 70–80% field capacity with 200 ml nutrient solution (KNO_3_ 9.89 mmol, (NH_4_)_2_SO_4_ 1.29 mmol, MgSO_4_ 2.08 mmol, KH_2_PO_4_ 2.57 mmol and FeSO_4_ 0.20 mmol per liter, pH 5.8) added to each pot every seven days. Healthy plants with a similar physical size at the budding stage were selected for the NaCl treatments, and those plants that survived the NaCl challenge were measured further.

Two transgenic potato lines (T3 and T13) had the highest expression of the *AtHKT1* gene, three had a moderate expression (T1, T2 and T5), and the remaining two had a low expression (T11 and T15). These transgenic lines, along with the non-transgenic control, were tested under four NaCl treatments: 0, 50, 100, and 150 mmol L^− 1^ NaCl. These salt levels were maintained by adding 1500 ml NaCl (pH 6.0) and 500 ml nutrient solution (pH 5.8) to each pot every seven days and a daily watering at 500 ml to maintain the vermiculite moisture at 70–80%. NaCl irrigation was started from potato initial flowering stage and continued for 30 days. The entire experiment with three replications was repeated three times, using three planting dates from April through August in 2013.

At each of the three runs, the treatments with three replicates were completely randomized. In each replicate, each treatment had 10 pots, and one plant per pot, resulting in a total of 960 pots (4 stress-treatments × 3 replicates × 8 lines × 10 pots each). The relative humidity in the greenhouse was at 70 to 75%, and the day/night temperatures were 25/15 °C. The photon flux density was 47.25 μmol m^− 2^ s^− 1^ and the day/night photoperiod was 14/10 h. At day 30 of NaCl treatments, the Na^+^ content in vermiculite was tested (Additional file 1: Table S1) and the following measurements were taken for each plant: plant height, stem thickness expansion rate, leaf area expansion rate, PN, Tr, Gs, Ci, total chlorophyll, proline, soluble sugar contents, RWC, MDA, electrolyte leakage, SOD, CAT and POD. These measurements were taken following the protocols previously described [49]. The leaves was used to test the K^+^ and Na^+^ contents. The roots were sampled to determine the root activity. The K^+^ and Na^+^ contents were measured as described by Ghars [52].

The root activity was determined using the triphenyl tetrazolium chloride (TTC) method [49]. The survival rate was counted as the ratio of the number of plants that survived the NaCl treatments to the total number of plants before the NaCl treatments at day 65 after the NaCl treatments and. At 90 days after seedling emergence (full maturity), the tubers, shoots, and roots were hand-harvested and weighed for each plant. The plant sample dry weights were determined after being oven-dried at 70 °C to a constant weight.

### Genetic stability analysis and quantification of salt tolerance

Plant genomic DNA was extracted from 0.1 g leaves of the control and all the transgenic plants. The *AtHKT1* gene was amplified using PCR techniques. All the methods were the same as those described in the above.

To quantify the degree of tolerance to NaCl, we calculated evaluation indices for all plant lines using an integrated cluster analysis in which standard deviation coefficients were integrated with allocation weighted values. The detailed methodology was described previously [49]. In brief, an evaluation index was developed using the following three steps:

(i) A subordinator function was formed and used to standardize the measured raw data of each parameter

1 $$ \mu \left({X}_{ij}\right)=\frac{\left(\mathrm{Xij}\hbox{-} \mathrm{Xmin}\right)}{\left(\mathrm{Xmax}\hbox{-} \mathrm{Xmin}\right)} $$

2 $$ \mu \left({X}_{ij}\right)=\frac{\left(\mathrm{Xmax}\hbox{-} Xij\right)}{\left(\mathrm{Xmax}\hbox{-} \mathrm{Xmin}\right)} $$

where *j* is the variable of six plant-growth traits, including daily gain of plant height, leaf area expansion rate, stem thickness expansion rate, plant tissue water content, tuber yield, and plant survival rate in salt treatment; *i* is the genotype; *Xij* is the value of the *j*th variable of the *i*th genotype; Xmin and Xmax are the minimal or maximal value among the X*ij* values for the *j*th variable, respectively. Equation (1) was to calculate positive correlation between a particular growth variable and salt tolerance, while eq. (2) was to calculate negative correlation.

(ii) A weighted coefficient was expressed by the standard deviation coefficient for each of the six plant-growth traits, as follow:

3 $$ Vj=\frac{\sqrt{\sum \limits_{i=1}^n{\left( Xij-\overline{Xj}\right)}^2}}{\overline{Xj}} $$

4 $$ Wj=\frac{Vj}{\sum \limits_{j=1}^m Vj} $$

where *n* represents the number of genotypes, and *m* represents the plant growth traits.

(iii) An integrated evaluation index was generated using the equation below:

5 $$ \mathrm{D}=\sum \limits_{j=1}^m\left[\mu (Xij)\times Wj\right]\kern0.6em \mathrm{j}=1,2,\dots, \mathrm{m} $$

where D represents the integrated evaluation index that quantifies the response of the potato genotypic lines to the different levels of salt stress [49].

### Statistical analysis

The data from the three runs of experiments were pooled together in the analysis, as the initial analysis showed that there were no significant treatment by run interactions for most of the variables measured, and the three runs of the experiment resembled the trend of treatment effects. All the data were analyzed using Tukey’s HSD test with the SPSS package (SPSS Software, 19.0, SPSS Institute Inc., USA). Cluster analysis and the quantitative assessment indices were used to categorize the seven genetic lines into the three levels of salt tolerance: low, medium, and high. The significances among treatments in the ANOVA or between the three levels of salt tolerance were determined at *P* < 0.05. The salt tolerance was assessed using parameters, including plant morphological and physiological traits, enzyme activities, and yield-related variables. The contributions of *AtHKT1* gene expression, salt tolerance evaluation index and plant physiochemical parameters to the overall variance were further analyzed using principal component analysis (PCA) and a two-dimensional PCA-biplot was generated to graphically demonstrate PC1 and PC2 scores. The angles between two vectors and the origin represent their relationship, with acute angles indicating positive correlations, right angles indicating no correlation, and obtuse angles indicating a negative correlation.

## Additional files

Additional file 1: **Table S1.** Na^+^ contents of vermiculite after 30 days NaCl treatment. (DOCX 16 kb)

Additional file 2: **Table S2.** Principle component analysis (PCA) total variance explained. (DOCX 17 kb)

Additional file 3: **Figure S1.** Schematic diagram and verification of the expression vector pAtHKT1. (JPG 323 kb)

Additional file 4: **Figure S2.** Formation of transgenic plants of the potato cultivar ‘Shepody’. (JPG 418 kb)

Additional file 5: **Figure S3.** PCR and PCR-Southern verification of transgenic plants. (JPG 299 kb)

Additional file 6: **Figure S4.** Verification of transgenic plants by reverse transcription polymerase chain reaction assay (RT-PCR) of *AtHKT1* gene. (JPG 187 kb)

Additional file 7: **Figure S5.** Growth characteristics (stems, leaves, and roots) of the transgenic potato line T3 (as an example) in comparison with the non-transgenic control (CK) plants under NaCl stress (0, 50, 100, 150mmol L^-1^). (JPG 357 kb)

Additional file 8: **Figure S6.** Classified clustering diagram on salt tolerance of transgenic potato lines carrying the *AtHKT1* gene. (JPG 162 kb)

## Data Availability

All relevant data used and/or analyzed during the current study are available from the corresponding author upon request.

## Abbreviations

CAT: Catalase
FW: Fresh weight
Gs: Stomatal conductance
MDA: Malondialdehyde
Pn: Net photosynthetic rate
POD: Peroxidase
SOD: Superoxide dismutase
Tr: Transpiration rate
WUE: Water use efficiency
