# Supplementary material for: Improving salt tolerance in potato through overexpression of AtHKT1 gene
Source: BMC Plant Biol. 2019 Aug 16;19:357. doi: 10.1186/s12870-019-1963-z (PMC6697938; doi:10.1186/s12870-019-1963-z)
Supplement: Supplementary file 1 — Table S1. Na+ contents of vermiculite after 30 days NaCl treatment. (DOCX 16 kb) [file 12870_2019_1963_MOESM1_ESM.docx]

| **Table S1** Na^+^ contents of vermiculite after 30 days NaCl treatment | |
| --- | --- |
| NaCl concentration ( mmol L^-1^) | Na^+^ contents (mg g^-1^) |
| 0 | 0.27 ± 0.01 d |
| 50 | 0.77 ± 0.03 c |
| 100 | 2.03 ± 0.06 b |
| 150 | 3.24 ± 0.09 a |
| Each value ± standard error was obtained from three runs of four treatments with three replicates in each run. The different letters denote significant differences according to Tukey's HSD test at P < 0.05. | |
