# Supplementary material for: Improving salt tolerance in potato through overexpression of AtHKT1 gene
Source: BMC Plant Biol. 2019 Aug 16;19:357. doi: 10.1186/s12870-019-1963-z (PMC6697938; doi:10.1186/s12870-019-1963-z)
Supplement: Supplementary file 2 — Table S2. Principle component analysis (PCA) total variance explained. (DOCX 17 kb) [file 12870_2019_1963_MOESM2_ESM.docx]

**Table S2** Principle component analysis (PCA) total variance explained

|  | Initial eigenvalues | | | Extraction sums of squared loadings | | | Rotation sums of squared loadings | | |
| --- | --- | --- | --- | --- | --- | --- | --- | --- | --- |
| Component | Total | % of variance | Cumulative % | Total | % of variance | Cumulative % | Total | % of variance | Cumulative % |
| 1 | 17.279 | 71.994 | 71.994 | 17.279 | 71.994 | 71.994 | 17.016 | 70.901 | 70.901 |
| 2 | 4.225 | 17.605 | 89.598 | 4.225 | 17.605 | 89.598 | 4.487 | 18.697 | 89.598 |
| 3 | 1.033 | 4.304 | 93.902 |  |  |  |  |  |  |
| 4 | 0.433 | 1.803 | 95.705 |  |  |  |  |  |  |
| 5 | 0.280 | 1.169 | 96.874 |  |  |  |  |  |  |
| 6 | 0.187 | 0.781 | 97.655 |  |  |  |  |  |  |
| 7 | 0.131 | 0.547 | 98.201 |  |  |  |  |  |  |
| 8 | 0.112 | 0.468 | 98.669 |  |  |  |  |  |  |
| 9 | 0.084 | 0.352 | 99.021 |  |  |  |  |  |  |
| 10 | 0.066 | 0.274 | 99.295 |  |  |  |  |  |  |
| 11 | 0.051 | 0.214 | 99.509 |  |  |  |  |  |  |
| 12 | 0.029 | 0.121 | 99.630 |  |  |  |  |  |  |
| 13 | 0.022 | 0.093 | 99.724 |  |  |  |  |  |  |
| 14 | 0.017 | 0.072 | 99.796 |  |  |  |  |  |  |
| 15 | 0.013 | 0.053 | 99.849 |  |  |  |  |  |  |
| 16 | 0.009 | 0.036 | 99.885 |  |  |  |  |  |  |
| 17 | 0.008 | 0.034 | 99.919 |  |  |  |  |  |  |
| 18 | 0.006 | 0.024 | 99.943 |  |  |  |  |  |  |
| 19 | 0.005 | 0.021 | 99.964 |  |  |  |  |  |  |
| 20 | 0.003 | 0.014 | 99.977 |  |  |  |  |  |  |
| 21 | 0.003 | 0.011 | 99.988 |  |  |  |  |  |  |
| 22 | 0.001 | 0.006 | 99.994 |  |  |  |  |  |  |
| 23 | 0.001 | 0.004 | 99.998 |  |  |  |  |  |  |
| 24 | 0.000 | 0.002 | 100.000 |  |  |  |  |  |  |
