# Supplementary figures and images for: Improving salt tolerance in potato through overexpression of AtHKT1 gene
Source: BMC Plant Biol. 2019 Aug 16;19:357. doi: 10.1186/s12870-019-1963-z (PMC6697938; doi:10.1186/s12870-019-1963-z)

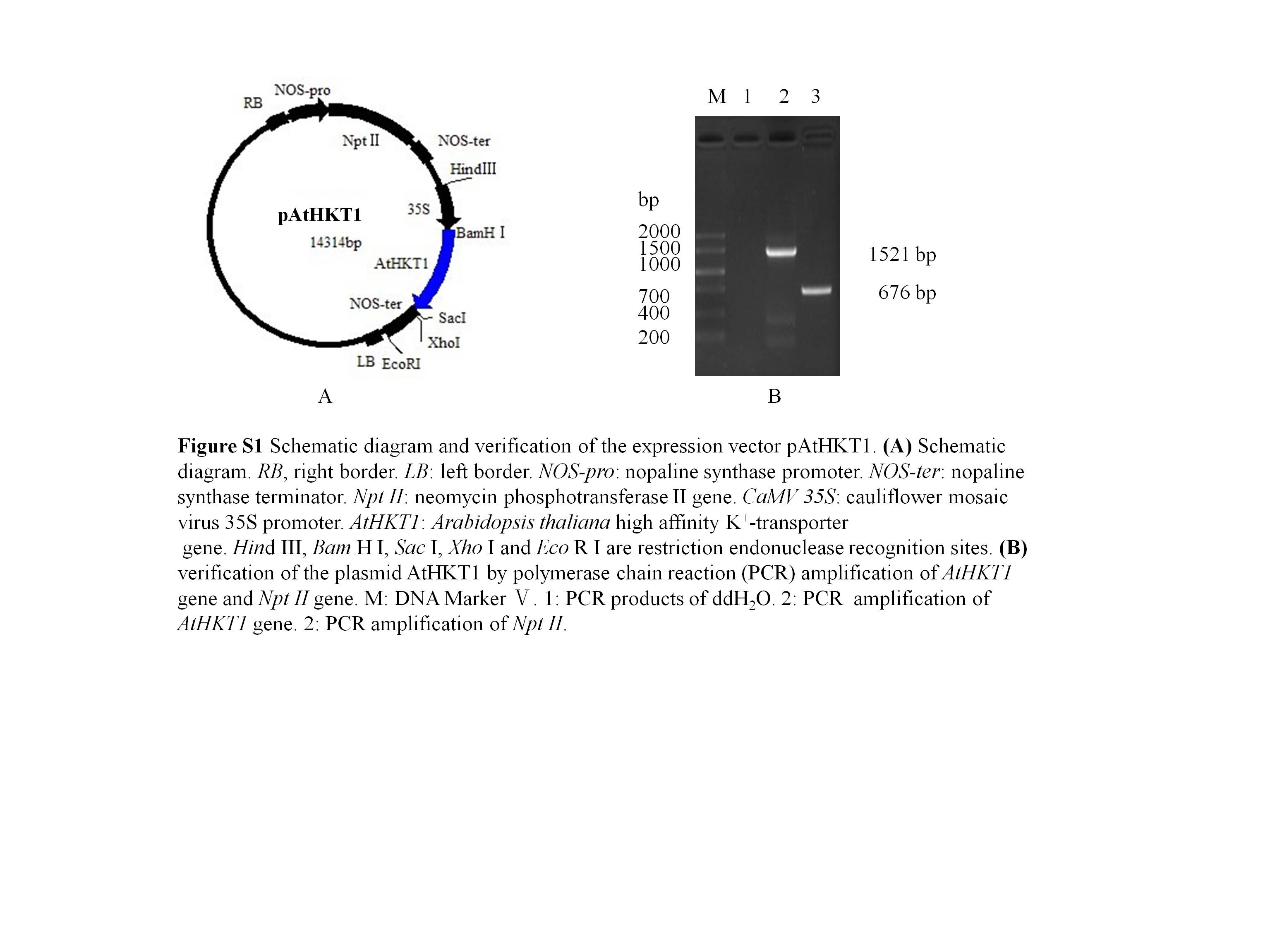

Supplement: Supplementary file 3 — Figure S1. Schematic diagram and verification of the expression vector pAtHKT1. (JPG 323 kb) [file 12870_2019_1963_MOESM3_ESM.jpg]

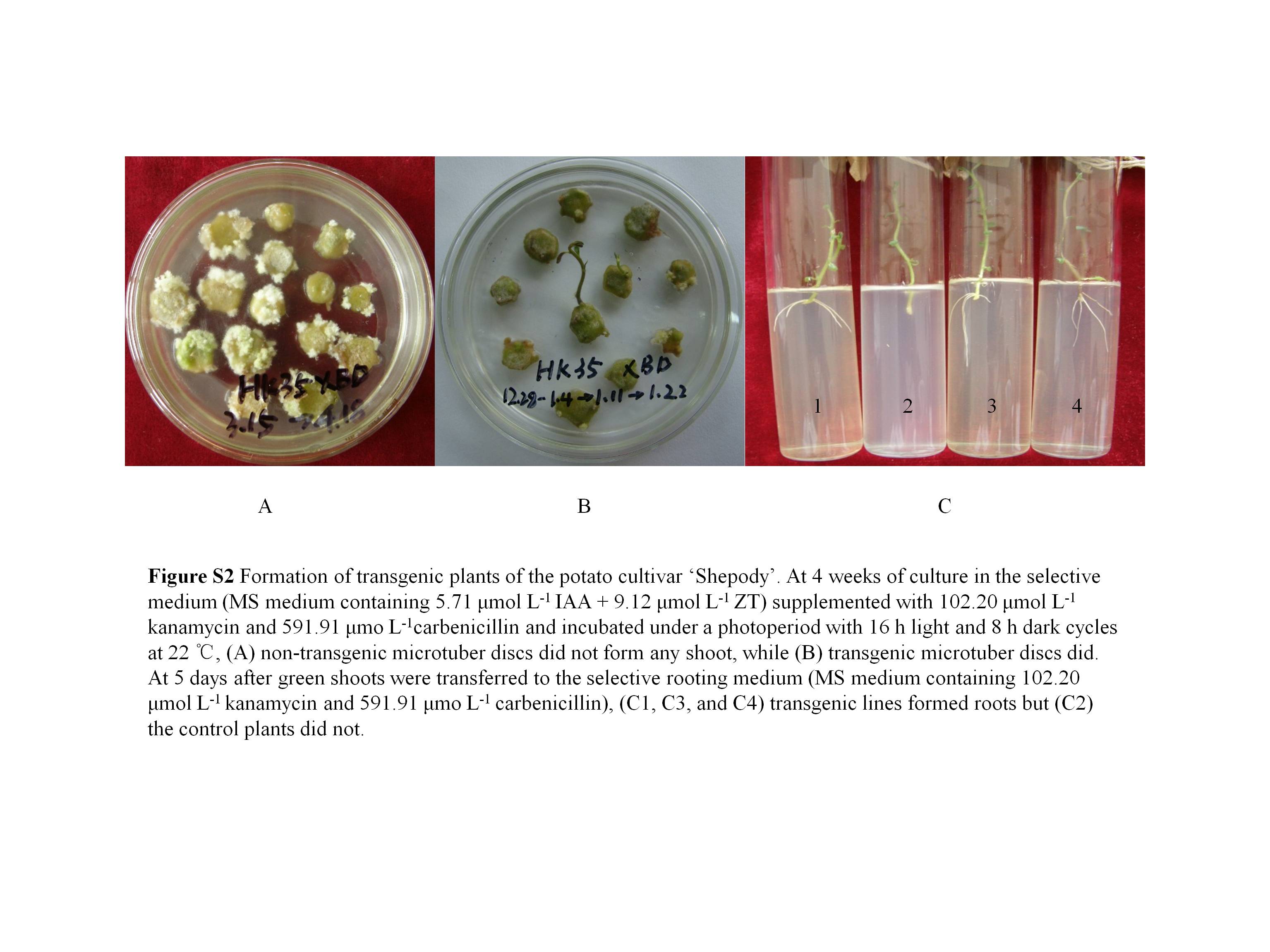

Supplement: Supplementary file 4 — Figure S2. Formation of transgenic plants of the potato cultivar ‘Shepody’. (JPG 418 kb) [file 12870_2019_1963_MOESM4_ESM.jpg]

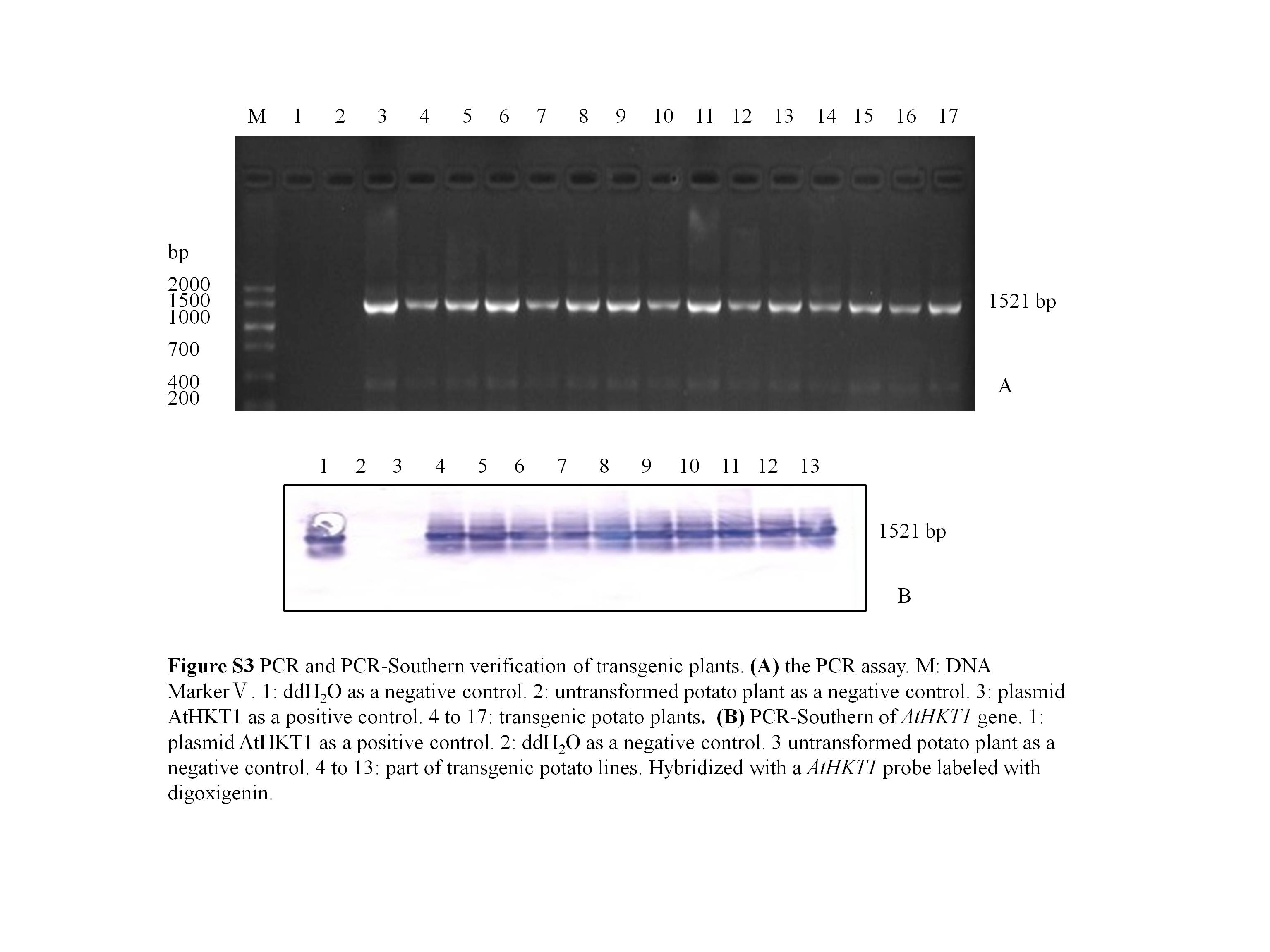

Supplement: Supplementary file 5 — Figure S3. PCR and PCR-Southern verification of transgenic plants. (JPG 299 kb) [file 12870_2019_1963_MOESM5_ESM.jpg]

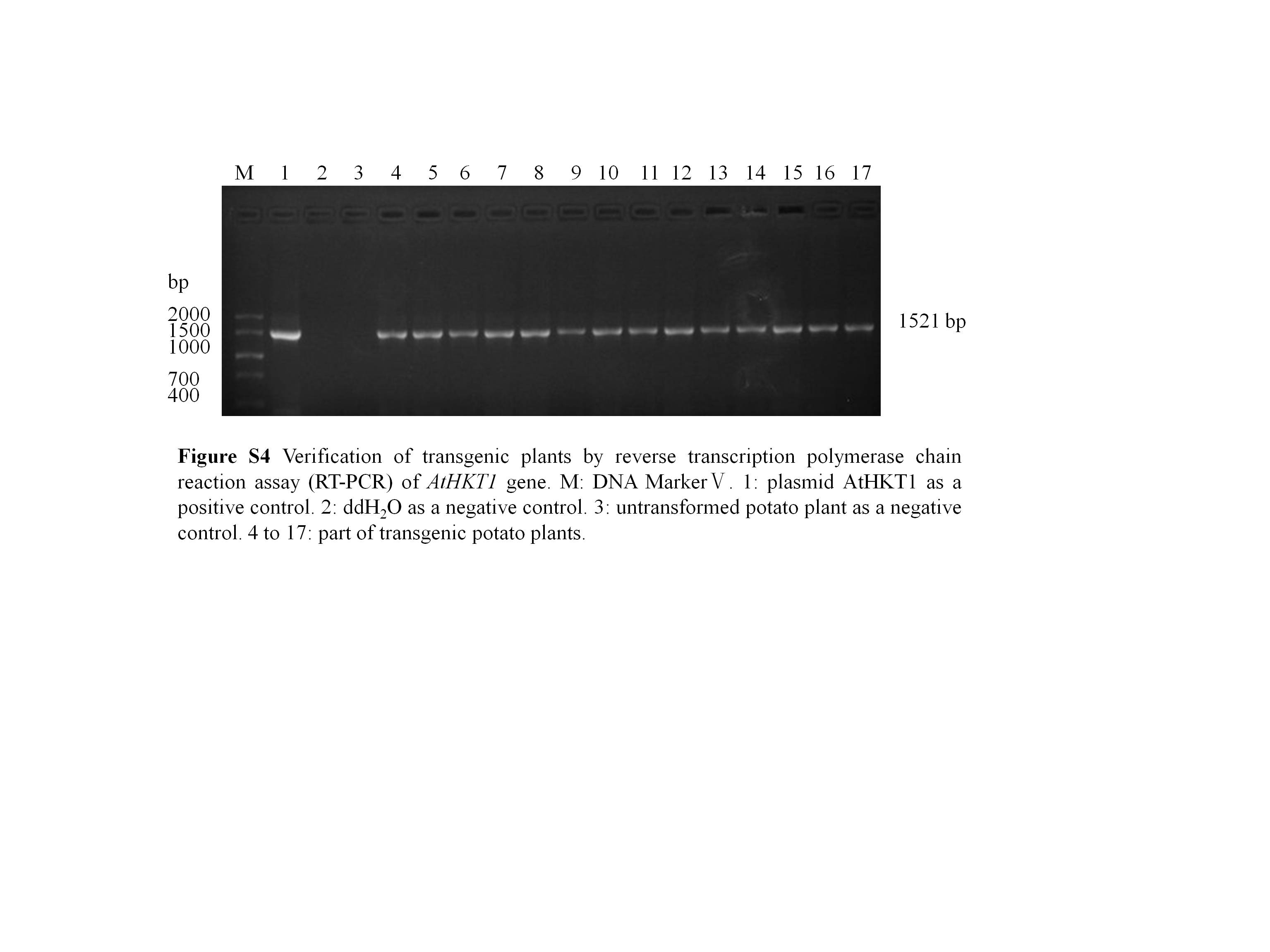

Supplement: Supplementary file 6 — Figure S4. Verification of transgenic plants by reverse transcription polymerase chain reaction assay (RT-PCR) of AtHKT1 gene. (JPG 187 kb) [file 12870_2019_1963_MOESM6_ESM.jpg]

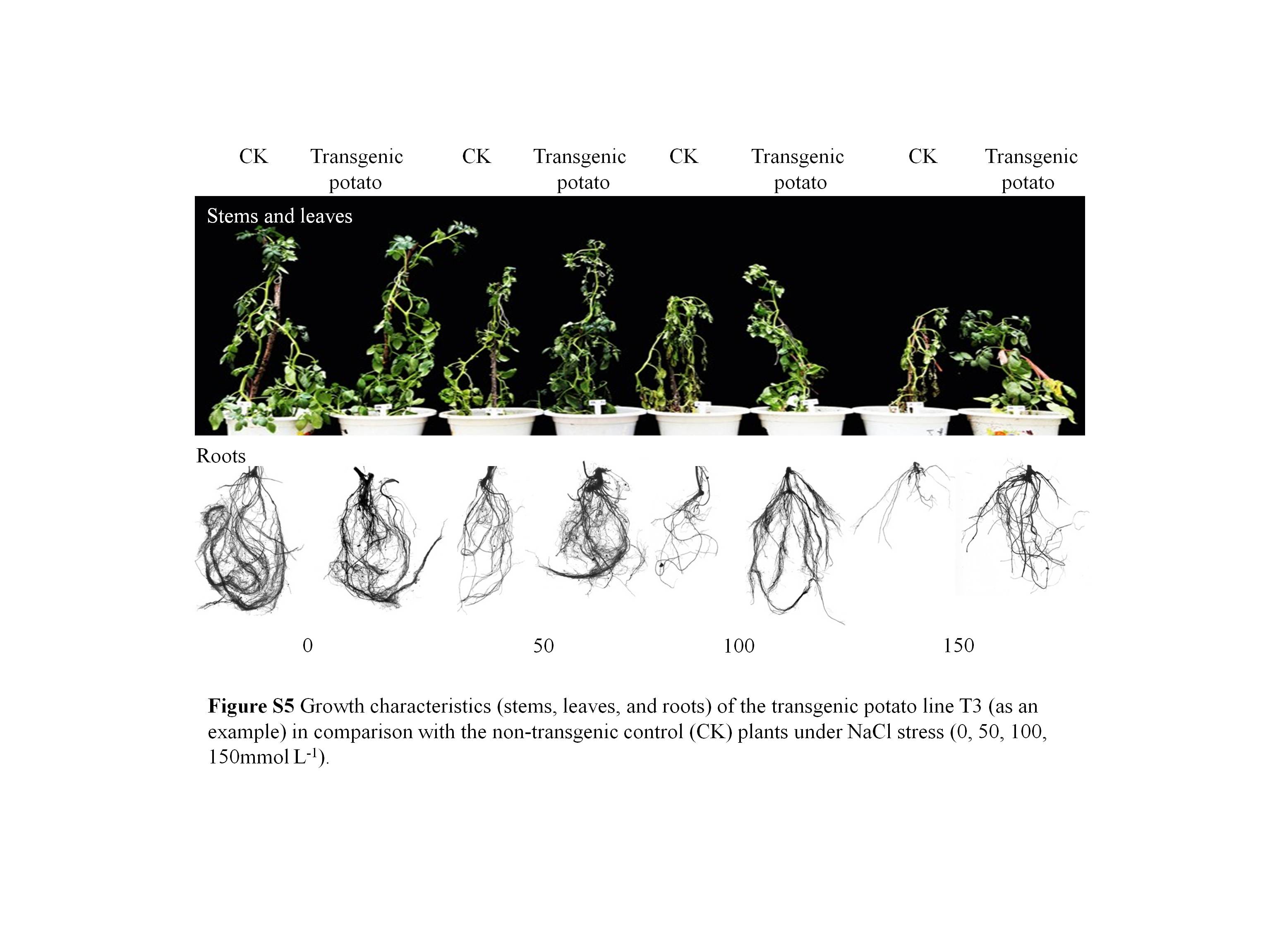

Supplement: Supplementary file 7 — Figure S5. Growth characteristics (stems, leaves, and roots) of the transgenic potato line T3 (as an example) in comparison with the non-transgenic control (CK) plants under NaCl stress (0, 50, 100, 150mmol L-1). (JPG 357 kb) [file 12870_2019_1963_MOESM7_ESM.jpg]

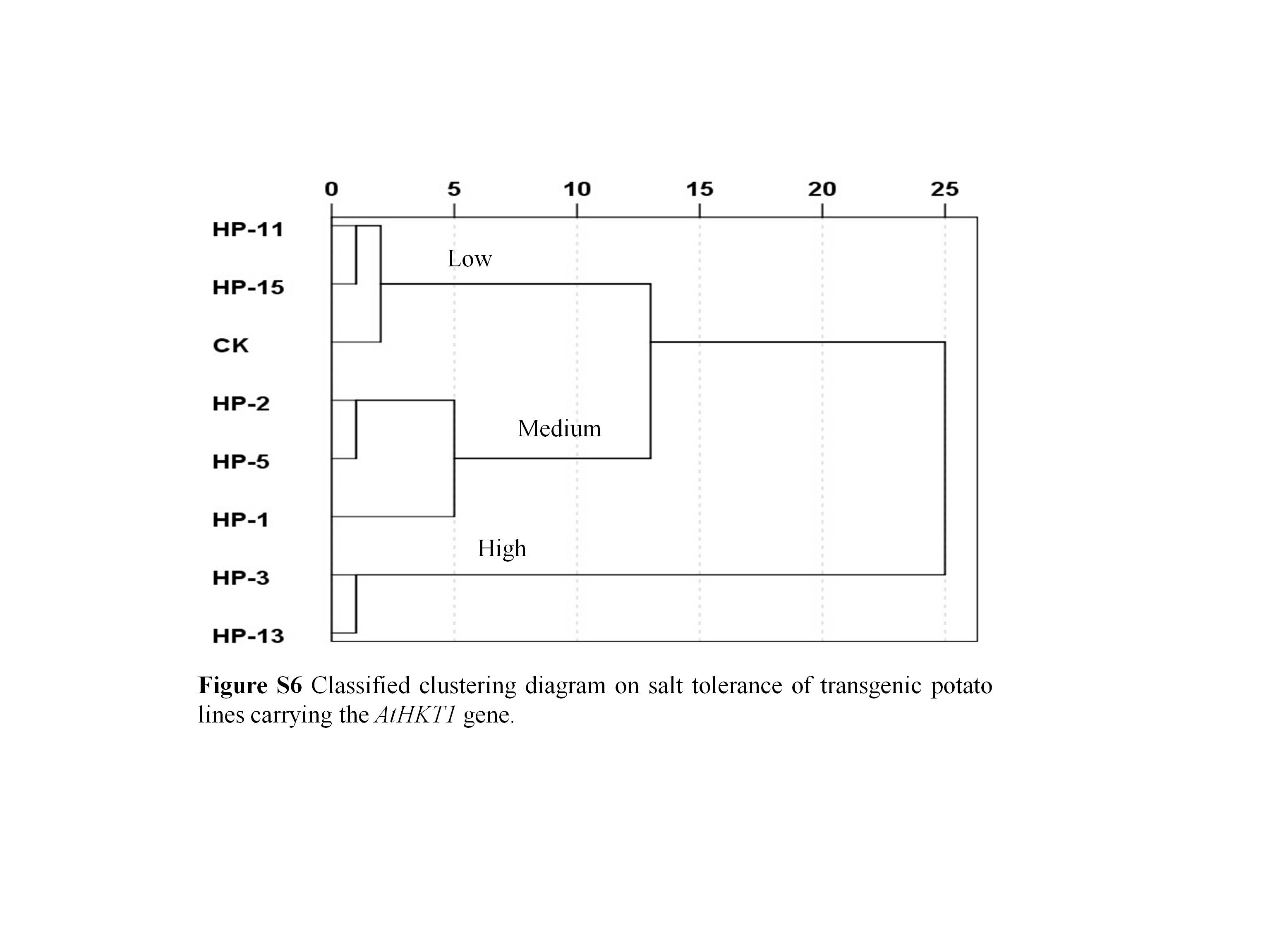

Supplement: Supplementary file 8 — Figure S6. Classified clustering diagram on salt tolerance of transgenic potato lines carrying the AtHKT1 gene. (JPG 162 kb) [file 12870_2019_1963_MOESM8_ESM.jpg]
